# New high throughput fully automated plasma GFAP immunoassay

**DOI:** 10.1002/alz.088246

**Published:** 2025-01-09

**Authors:** Miklos Szabo, Mike Salvati, Ben Schlichtmann, Jason Patzlaff, Dusten Unruh, Kara Curtis, Laura Mediger, Mikaela Nichkova‐Doseva

**Affiliations:** ^1^ Beckman Coulter Inc., Chaska, MN USA

## Abstract

**Background:**

The analytical performance characteristics for the plasma Glial Fibrillary Acidic Protein (GFAP) immunoassay currently under development on Beckman Coulter, Inc. Access2™ and DxI9000™ analyzers is described. Blood GFAP levels may be indicative of the extent of neurologic injury in diseases such as TBI and stroke and maybe used as a marker of disease progression in other diseases such as multiple sclerosis. Plasma GFAP outperforms its CSF counterpart in detecting AD pathology and correlation to the clinical stage of the disease

**Method:**

The prototype GFAP assay is a one‐step sandwich assay utilizing an anti‐GFAP mouse monoclonal (MAb) antibody/alkaline phosphatase conjugate and paramagnetic particles coated with a complementary anti‐GFAP mouse MAb. Sample and reactants are incubated, washed, then a chemiluminescent substrate is added. The light that is generated is directly proportional to the GFAP concentration in the sample. The assay time to first result is ∼30 minutes. 43 EDTA plasma samples, consisting of 19 Alzheimer’s Disease (AD) patient samples and 24 age‐matched healthy normal samples, were evaluated. A method comparison was performed between Access2™ and DxI9000™ platforms.

**Result:**

The prototype GFAP assay has a targeted analytical measuring range of 1.0 pg/ml to approximately 700.0 pg/ml (capable of measuring analyte up to 10,000 pg/ml). 100% of normal samples were quantified (90% measuring below 20.0 pg/ml), with low dose variability (<4.0% coefficient of variation). A comparison of the prototype GFAP assay on DxI9000™ against Access2™ gave a Passing‐Bablok slope of 0.94 (R=0.992) and an intercept of 0.37 pg/ml. Concordance analysis shows excellent agreement between the Access2™ and DxI9000™ assays. The dose ratio of the median of AD over the median of normal samples for Access2™ and DxI9000™ is 2.31 and 2.41, respectively, with AD patient median dose values of 14.3 pg/ml and 14.6 pg/mL, respectively, and normal sample median dose values of 6.2 pg/ml and 6.0 pg/mL, respectively.

**Conclusion:**

The prototype GFAP assay provides fast, highly sensitive and precise results in an automated immunoassay on the Beckman Coulter Access2™ and DxI9000™ platforms. The prototype plasma GFAP assay could be used to both diagnose and monitor various neurodegenerative diseases such as AD.

